# Does lock-down of Wuhan effectively restrict early geographic spread of novel coronavirus epidemic during *chunyun* in China? A spatial model study

**DOI:** 10.1186/s12889-021-10837-2

**Published:** 2021-04-29

**Authors:** Yi Hu, Lingcai Kong, Tong Yao, Xinda Chen, Wei Du

**Affiliations:** 1 Department of Epidemiology and Biostatistics, School of Public Health, Fudan University, Shanghai, 200032 China; 2 Department of Mathematics and Physics, North China Electric Power University, Baoding, 071003 China; 3 Key Laboratory of Environmental Medicine Engineering, Ministry of Education; School of Public Health, Southeast University, Nanjing, 210009 Jiangsu China

**Keywords:** Coronavirus, COVID-19, Geographic spread, Lock-down, *Chunyun*, China

## Abstract

**Background:**

Prior to Wuhan lock-down in 2020, *chunyun*, the largest population mobility on this planet, had begun. We quantified impact of Wuhan lock-down on COVID-19 spread during *chunyun* across the nation.

**Methods:**

During the period of January 1 to February 9, 2020, a total of 40,278 confirmed COVID-19 cases from 319 municipalities in mainland China were considered in this study. The cross-coupled meta-population methods were employed using between-city Baidu migration index. We modelled four scenarios of geographic spread of COVID-19 including the presence of both *chunyun* and lock-down (baseline); lock-down without *chunyun* (scenario 1); *chunyun* without lock-down (scenario 2); and the absence of both *chunyun* and lock-down (scenario 3).

**Results:**

Compared with the baseline, scenario 1 resulted in 3.84% less cases by February 9 while scenario 2 and 3 resulted in 20.22 and 32.46% more cases by February 9. The geographic distribution of cases revealed that *chunyun* facilitated the COVID-19 spread in the majority but not all cities, and the effectiveness of Wuhan lock-down was offset by *chunyun*. Impacts of Wuhan lock-down during *chunyun* on the COVID-19 spread demonstrated heterogenetic geographic patterns.

**Conclusion:**

Our results strongly supported the travel restriction as one of the effective responses and highlighted the importance of developing area-specific rather than universal countermeasures.

Contributions to the literature

- Chunyun, the largest population mobility on this planet, was a stimulus of the early geographic spread of COVID-19 in China.
- Domestic travel restriction served as one of the effective emergency responses to the COVID-19 outbreak.
- Area-specific countermeasures with particular relevance to many countries around the world that faced massive inter-city travel demands would include continuing restriction on population mobility.

## Background

Since early December 2019, an increasing number of atypical pneumonia have been reported in Wuhan [1], a city with a population of 11 million in the central part of China. In response to this outbreak, the Chinese Center for Disease Control and Prevention (China CDC) conducted an epidemiologic and etiologic investigation on December 31, 2019 [2], and found that human-to-human transmission occurred since the middle of December 2019 [3], and isolated and confirmed a novel strain of coronavirus on 7 January 2020 [4]. Along with the increasing number of COVID-19 cases in China, the geographic spread at a meta-population level (e.g. between cities in China, Asia-pacific regions, or northern hemisphere countries) has been reported [5].

When human-to-human transmission demonstrated a population health threat, use of restrictive measures such as isolation of cases and quarantine of contacts became one of the apparent emergency responses to the COVID-19 outbreak [6]. However, a substantial challenge has emerged when *chunyun,* the largest human migration on the planet, beginning on Jan 10, 2020 with billions of trips made for family reunions to celebrate the Spring Festival during the national holidays from January 24 to 31 [7]. Geographic spread of COVID-19 would have potentially be accelerated by *chunyun* under this circumstance, and therefore prevention and control of the COVID-19 infiltration into local communities across the nation would require immediate actions to restrict human movements. On January 23 and 24, 2020, the Chinese central government implemented the metropolis-wide lock-down of Wuhan and its surrounding satellite cities [3]. In addition to the Wuhan lock-down, the central government announced the extension of the national holidays, the Spring Festival, and set the back to work date as of February 10, 2020 (except Wuhan) [8].

In the face of this unprecedented threat and lack of effective countermeasures, the authorities invoked the lock-down of Wuhan as a means to interrupt the geographic spread of COVID-19 and expected to achieve the disease control goals [9]. Although several studies investigated the impact of city lockdown on the disease spread [10–12], population based evidence is inadequate regarding their individual roles of city lockdown and *chunyun* as well as their aggregated role on the spread of COVID-19 in real world settings. This study aimed to evaluate the effectiveness of Wuhan lock-down for preventing the spread of COVID-19 at an early stage, and examine whether the effectiveness would vary according to the presence or absence of *chunyun* and lock-down.

## Methods

### Data sources

Provincial Health Commissions in mainland of China, in collaboration with provincial and municipal CDCs, have validated, documented, and reported municipal-level incident numbers of COVID-19 suspected, confirmedly infected, recovered, and deceased individuals, respectively on a daily basis since January 2020 [13]. We included a total of 319 municipalities having at least one laboratory-confirmed case and ascertained the daily numbers of COVID-19 cases in each city from January 1 to February 9, 2020. These data were publically available and therefore this study was exempted for ethics approval by institutional review boards with respect to data collection, analysis and reporting. The study outcome was the laboratory-confirmed COVID-19 incidents.

Baidu Migration Index is a free data analytic platform using Baidu web search and Baidu news to present massive behavioral data among Baidu users, which has been frequently used to reflect population mobility in China [14]. We obtained Baidu Migration Index from January 1 to February 9, 2020 to quantify the daily number of travelers between pair-wise cities. The specific number of travelers from city *i* to *j* at day *t*, *X*_*i*, *j*, *t*_, was calculated as follows:

1 $$ {X}_{i,j,t}={p}_{i,j,t}\ast \frac{No\_ wh}{p_{wh}} $$

where *p*_*i*, *j*, *t*_ is the migration index from city *i* to *j* at day *t*, *No* _ *wh* is the number of travelers leaving Wuhan during January 10 to January 19, 2020 (prespecified as 4.10 million [15]), and *p*_*wh*_ is the sum of traveling index from Wuhan to all the other cites during the same period.

### Statistical analysis

We used the cross-coupled meta-population (epidemic) model with an addition of population mobility matrix to complement the standard Susceptible-Exposed-Infectious-Removed (SEIR) model considering the geographic spread of COVID-19 between cities across the nation:

2 $$ \frac{d{S}_i}{dt}=-{\beta}_t{S}_i{\sum}_{j=1}^n\frac{\varphi_{i,j,t}{I}_j}{N_i} $$

3 $$ \frac{d{E}_i}{dt}={\beta}_t{S}_i{\sum}_{j=1}^n\frac{\varphi_{i,j,t}{I}_j}{N_i}-\alpha {E}_i $$

4 $$ \frac{d{I}_i}{dt}=\alpha {E}_i-\gamma {I}_i $$

5 $$ \frac{d{R}_i}{dt}=\gamma {I}_i $$

where *S*_*i*_, *E*_*i*_, *I*_*i*_, and *R*_*i*_ are the numbers of susceptible, exposed, infectious, and recovered individuals, respectively, and *N*_*i*_ is the total population size of city *i*, *β*_*t*_ is the transmission parameter (we assumed it is the same across all cities) at time *t*, *φ*_*i*, *j*, *t*_ is the proportion of individuals moving to city *i* from city *j* at time *t*, *α* is the latent rate, and *γ* is the recovery rate. For the convenience of model fitting, we added another compartment (*K*) to the above equations to keep track of cumulative incidence as follows:

6 $$ \frac{d{K}_i}{dt}=\alpha {E}_i $$

Of meta-population models, there are mainly two types: cross-coupled and mobility models, in which individuals in all states move. There is, however, no advantage of one over the other [16]. Model fitting was achieved by treating the differential equation (Eq. 6) as representing the mean number of cumulative cases per day in China during the study period. Parameter inference was achieved by least square (LS) estimation using L-BFGS-B optimization with the *optim()* function in the R statistical language (R Core Team, 2020). Uncertainty was analyzed using parametric bootstrap method. A total of 1000 simulations from the model (Eq. 6) was firstly generated using the LS estimates of the parameters. Each simulated dataset was then re-fitted into the model to construct a joint sampling distribution of the parameters, with 95% confidence interval estimated using the lower 2.5% and upper 97.5% quantiles.

The instantaneous basic reproductive number (*R*0_*t*_) was calculated by *β*_*t*_/*γ*. We then simulated the probable course of the COVID-19 spread conditioned on different modelling scenarios (ESRI Inc., 2020), including the presence of both *chunyun* and lock-down (baseline, the real world scenario); lock-down without *chunyun* (scenario 1); *chunyun* without lock-down (scenario 2); and the absence of both *chunyun* and lock-down (scenario 3).

## Results

**Fig. 1:**
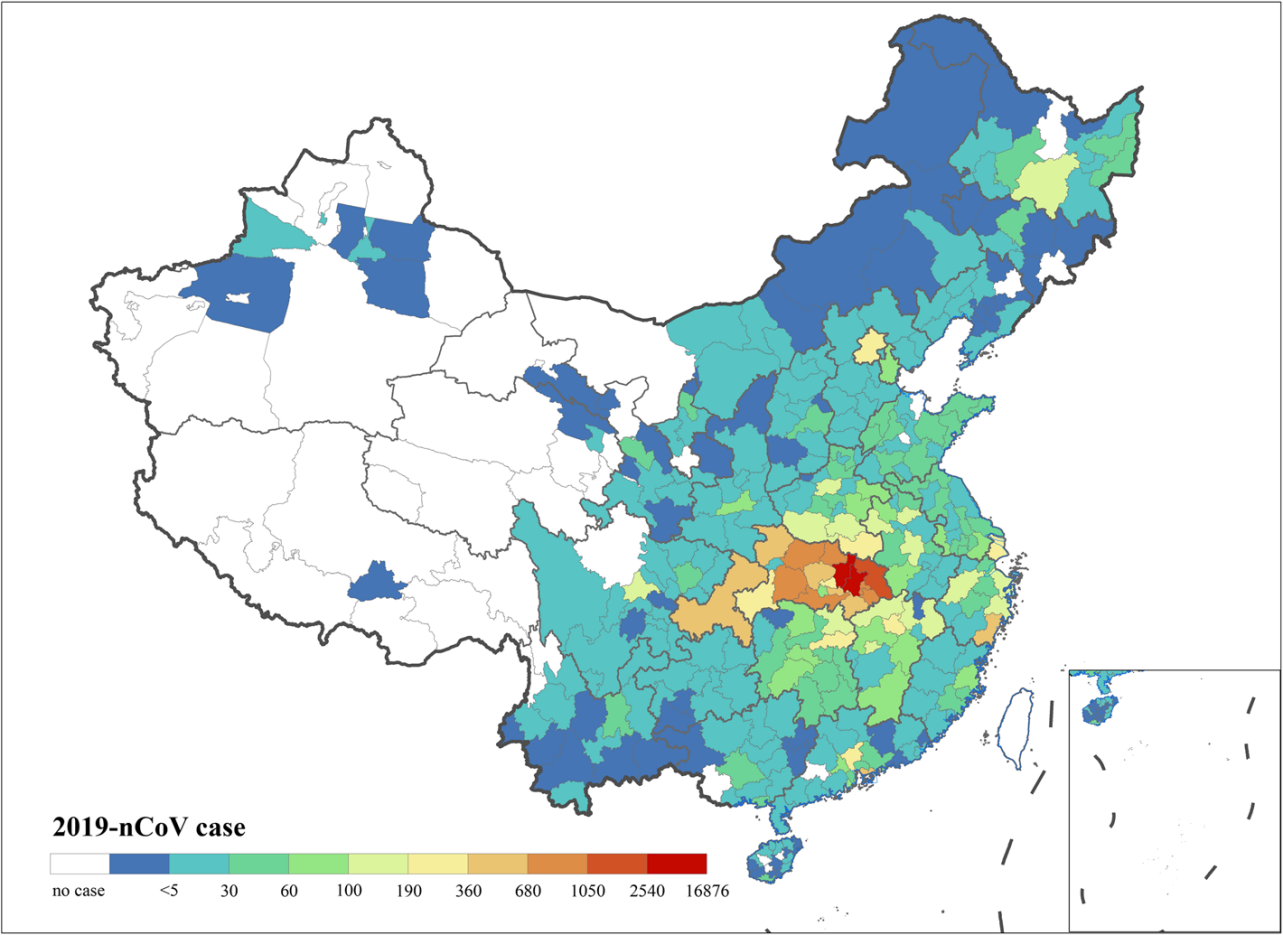
Cumulative cases of COVID-19 in China on February 9, 2020. This figure was produced in ArcGIS 10.4.1 (ESRI, Redlands, CA, USA) using shape files representing China’s municipal-level administrative units freely downloaded from Resource and Environment Science and Data Center (http://www.resdc.cn/data.aspx?DATAID=201)

**Fig. 2:**
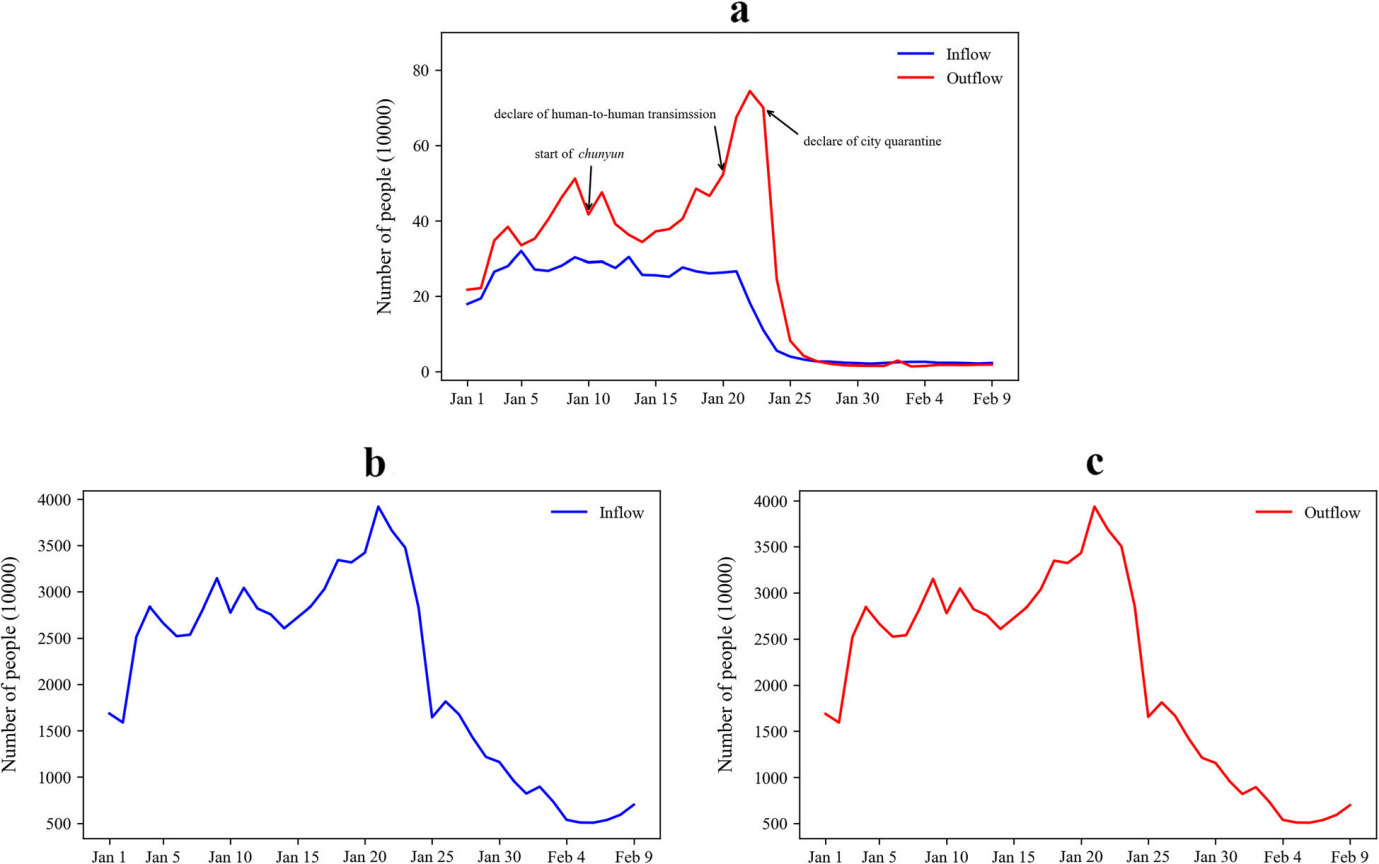
Population migration in Wuhan (**a**) and China (**b** and **c**), depicted from Dr. Hu’s own work

During the period of January 1 to February 9, 2020, a total of 40,278 confirmed COVID-19 cases from 319 municipalities in mainland China were reported (Fig. 1). Across China, the population mobility had been increasing since the start of *chunyun* and reached the greatest on January 21 and then decreased afterward (Fig. 2). While the patterns of population inflow and outflow in China were similar, these patterns for population inflow to and outflow from Wuhan presented in different ways. The population outflow from Wuhan showed a generally increasing trend up to January 22 whereas the population inflow to Wuhan remained almost constant. It is noteworthy for Wuhan that the population outflow was always greater than the population inflow until January 26 (shortly after the announcement of lock-down). Change of population outflow from Wuhan was also noteworthy for the sharp increase after announcement of human-to-human transmission of COVID-19 on January 20.

**Fig. 3:**
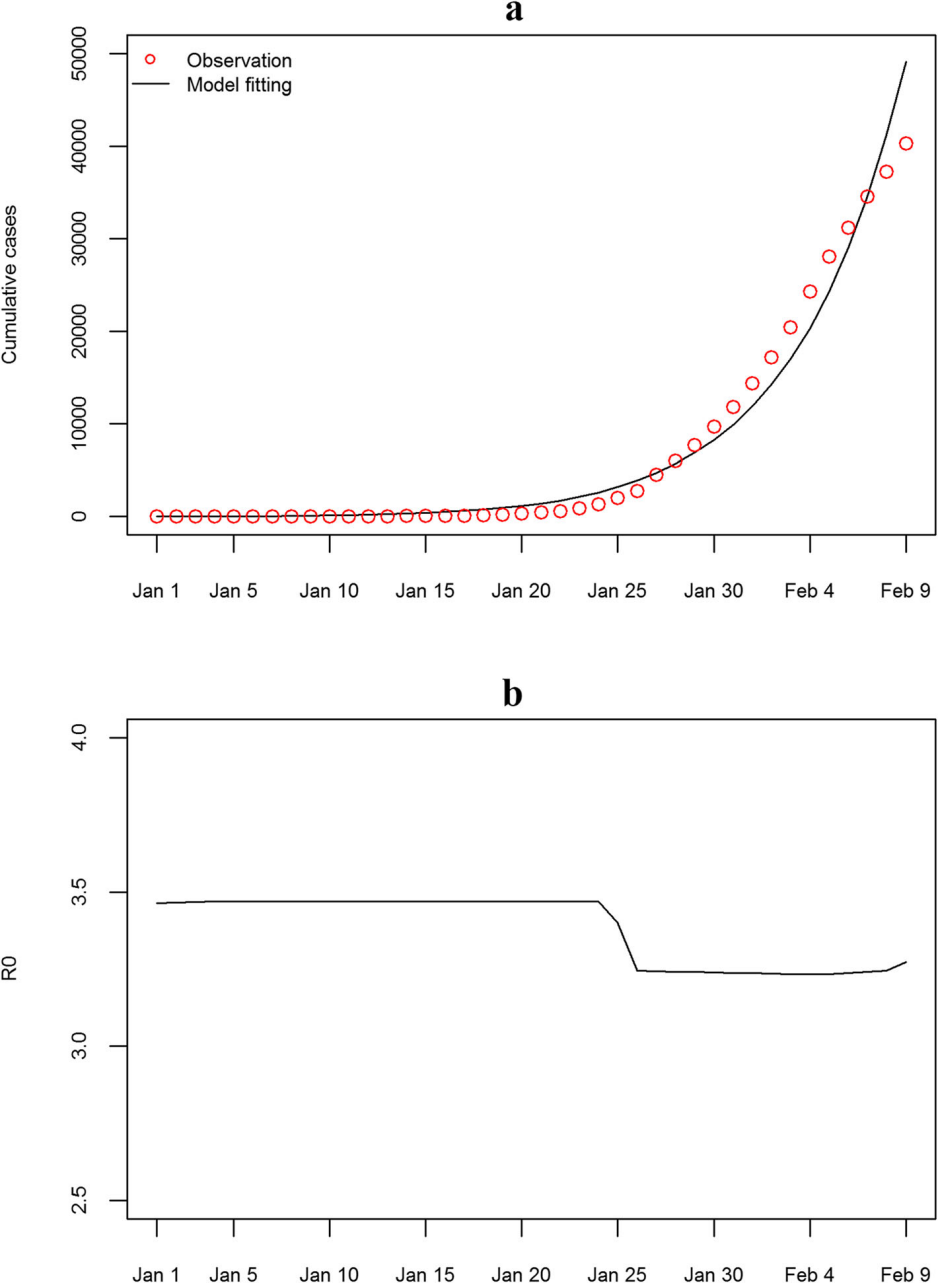
**a**, Model fitting with the cumulative cases of COVID-19 in China; **b**, instantaneous basic reproductive number (*R*0_*t*_) of COVID-19 during Jan 1 to Feb 9, 2020

Figures 3a and b illustrates a reasonably good model fit and the time-varying estimates of basic reproductive number (*R*0_*t*_) of COVID-19 during the study period. Although we assumed *R*0_*t*_ varies over time, it stayed at the same level from January 1 to 25 and fell from 3.47 to 3.24 from January 26 onwards. Note that it slightly increased to 3.27 on February 9. The modelled latent and infectious time of COVID-19 was 6.11 days (95%CI: 3.13, 10.63) and 3.26 days (95%CI: 1.06,5.16), respectively.

Table 1 displays cumulative number of COVID-19 cases in China during the study period under different modelling scenarios. Under Scenario 1, the COVID-19 epidemic would have resulted in 3.84% less cases than the baseline by February 9, indicating that *chunyun* facilitated the spread of this infectious disease. Compared with the baseline scenario, scenario 2 would have produced 32.46% more COVID-19 cases, demonstrating the protective effectiveness of Wuhan lock-down. Under Scenario 3, the COVID-19 epidemic would have resulted in 20.22% more cases than the baseline in the absence of *chunyun* and lock-down.

**Table 1:** Total number of COVID-19 cases in China under different scenarios

Scenario	Description	Number of cases on Feb 9, 2020
Baseline	*Chunyun* (yes), lock-down (yes)	48,637
1	*Chunyun* (no), lock-down (yes)	46,769 (**−3.84%**)
2	*Chunyun* (yes), lock-down (no)	64,424 (**32.46%**)
3	*Chunyun* (no), lock-down (no)	58,473 (**20.22%**)

Percentage change (%) was based on the baseline and highlighted in bold

**Fig. 4:**
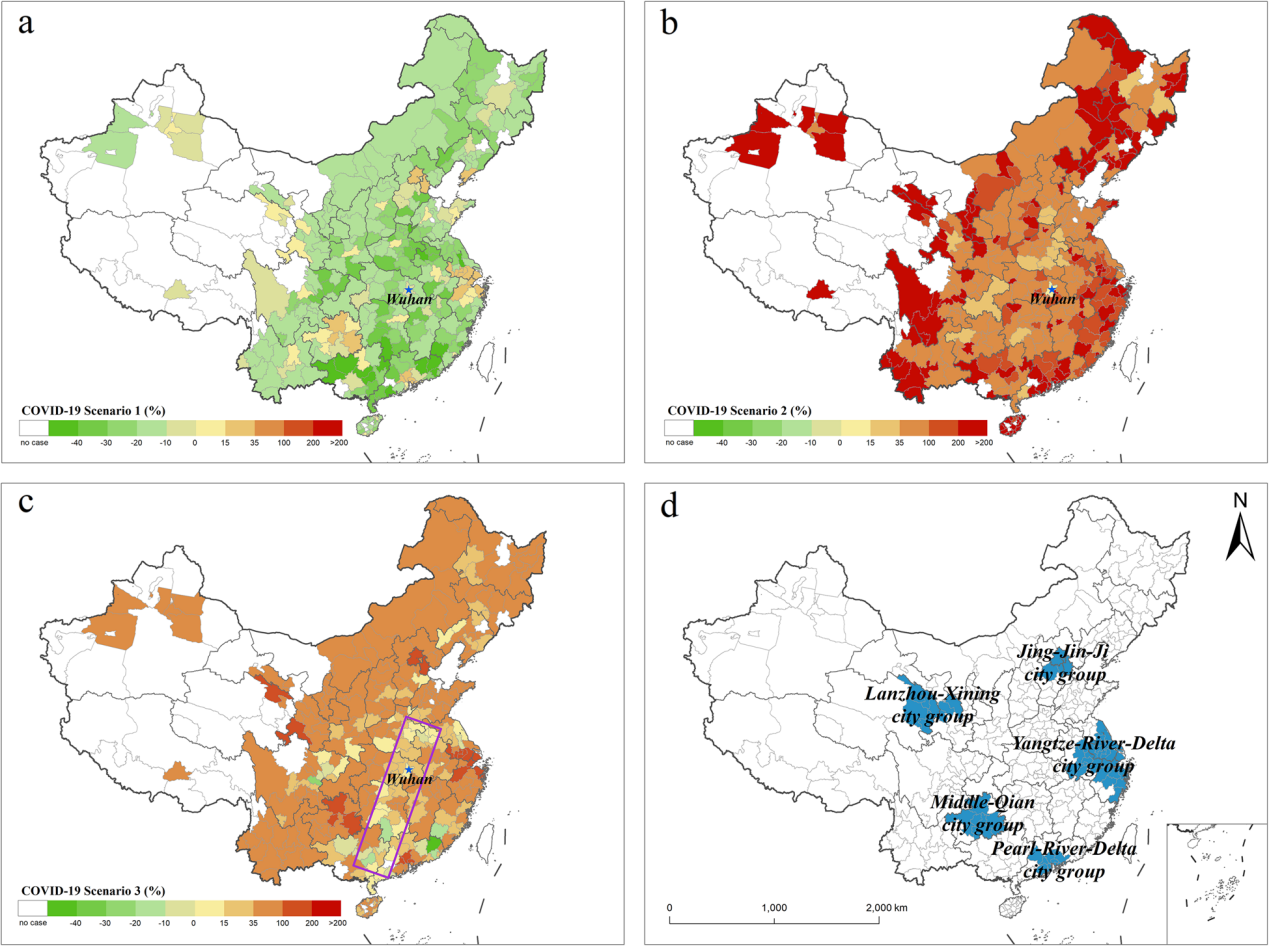
Geographical distribution of change (quantified by percentage) of cumulative COVID-19 cases in comparison with baseline (the presence of both *chunyun* and lock-down) by February 9, 2020, depicted from Dr. Hu’s own work. **a**, scenario 1 (lock-down without *chunyun*); **b**, scenario 2 (*chunyun* without lock-down); **c**, scenario 3 (the absence of both *chunyun* and lock-down); **d**, location of urban agglomeration. This figure was produced in ArcGIS 10.4.1 (ESRI, Redlands, CA, USA) using shape files representing China’s municipal-level administrative units freely downloaded from Resource and Environment Science and Data Center (http://www.resdc.cn/data.aspx?DATAID=201)

Figure 4 demonstrates the geographic distribution of change in cumulative COVID-19 cases comparing different scenarios with the baseline scenario (the presence of both *chunyun* and lock-down) by February 9, 2020. Under scenario 1 (Fig. 4a), the majority of cities showed a relatively sharp change in case reduction despite the nuance expression in a few populous cities, indicating that *chunyun* was not a common stimulus for the COVID-19 spread across the nation. Under scenario 2 (Fig. 4b), all the cities would have had greater number of cases in the absence of lock-down, in particular, those in northeast, south and west China would have an increase over 100%, indicating the protective effect of Wuhan lock-down on preventing additional disease penetration towards all the other cities in China. Under scenario 3 (Fig. 4c), the protective effect of Wuhan lock-down varied in space and was offset by the presence of *chunyun*, especially for those corridor cities near Wuhan. Note that areas with over 100% increase of cases (in dark red color) under this scenario were mainly located within the five city groups shown in Fig. 4d.

## Discussion

In this retrospective analysis of 40,278 confirmed COVID-19 cases in China, we modelled 3 exposure scenarios using publically available data reported on a daily basis by local public health authorities. These scenarios differed in the exposure to *chunyun*, the largest population mobility on the earth, and Wuhan lock-down, the unprecedented control of 11 million people’s movement in response to the rapid spread of COVID-19 from the city. Of the simulations of three exposure scenarios, the lock-down of Wuhan remarkably demonstrated the protective effects by preventing 32.46% COVID-19 incidents by February 9, 2020, whereas *chunyun* contributed towards the observed geographic spread and would have produced 3.84% more cases by the same period. Although the impact of the presence of both *chunyun* and lock-down of Wuhan on the COVID-19 spread was heterogeneous in space, the majority of cities had been protected with risk of a subsequent outbreak mitigated by the lock-down in spite of the *chunyun* offset. In addition to previous findings allowing for the effects of fleeing population [11], flight restriction [12], and limited medical resources [10], we further demonstrated the individual role for *chunyun* and its combined role with the lock-down of Wuhan in comparison with the absence of both events. These findings complemented the growing evidence of impact of population mobility on the geographic spread of COVID-19, justified the invocation of Wuhan lock-down instead of simply claiming the precautionary principle, and provided additional vigilance against inter-city human movement at the critical early phase for next-stage informed decision making with respect to COVID-19 prevention and control in the communities.

In this study, we also investigated the epidemiological characteristics of COVID-19 outbreak in China. We first estimated its instantaneous *R*0_*t*_, and found a decrease from 3.47 to 3.24 on January 26, which was probably due to the observed decrease in population mobility (Fig. 2). In addition, our estimate of latent and infectious periods were 6.11 and 3.26 days, respectively, with the former being comparable with the existing clinical studies [17, 18] and the latter, being rarely reported. Compared with the previously reported basic reproductive number (*R*0) which were fixed and ranged from 2.24 to 3.80 [3, 18–20], our estimate was derived from a time varying model, which was somewhat more reliable as we have taken into account a larger sample size as well as population mobility between cities that would potentially affect both the contact rates between individuals and subsequently the estimation of COVID-19 spread. Notwithstanding the decrease of *R*0_*t*_, there was an increasing trend on February 9 which corresponded to the time when migrants returned to study or work. Considering the contribution of *chunyun* towards geographic spread of COVID-19, this rebound trend of *R*0_*t*_ implied that the epidemic would perhaps spread more rapidly and move into the next stage of becoming endemic. Therefore, continuing efforts with rigorous prevention and control measures are required and expected to be strictly implemented in the communities including social distancing and home quarantine.

We estimated the COVID-19 occurrence under three scenarios and their respective changes in relation to the baseline incidents for each city. We found that there was evidently spatial heterogeneity in effects of *chunyun* and/or Wuhan lock-down. In principle, *chunyun* contributed towards the COVID-19 spread in China (Table 1) but not specifically for each locality (Fig. 4a), while the Wuhan lock-down restricted the subsequent spread towards the other cities across the nation (Table 1, Fig. 4b). In the absence of both *chunyun* and lock-down (Fig. 4c), more evident reduction in COVID-19 incidents would have occurred for five major urban agglomerations. These urban agglomerations (Fig. 4d) consist of two kinds of city clusters, i.e., well-developed megacities, and the others nested in the undeveloped regions of China. There is an obvious speculation that these clusters would experience similar outgoing and ingoing travel demands all year round, and therefore the effects of *chunyun* would be nuance. It is noteworthy that corridor cities near Wuhan (Fig. 4c) did not benefit much from the Wuhan lock-down as its protective effect was offset by *chunyun*. These corridor cities would perhaps become a case reservoir prior to the lock-down of Wuhan. Therefore, these corridor cities should be given a priority for continuing efforts in allocation of healthcare resources. Additional strategies should be developed based on their area-level characteristics.

Evidence of comparative effectiveness of large-scale lock-down on 11-million populations is rare because the outbreak of contagious disease of this kind is highly unusual and so is to have reliable national data. Using a larger sample size than previous studies and clear evidence of exposure, our findings are somewhat robust. However, there are some limitations. First, the use of deterministic SEIR model would be acceptable given the successful control of COVID-19 in China [21]. Yet, our analysis did not consider the under-reporting of cases during the study period, due in part to the pre-symptomatic transmission and asymptomatic individuals are neglected [22], which may result in an underestimation bias of our results. Iterated filtering algorithm such as Maximum Likelihood Estimation or Bayesian approach could be another option [23], especially in other settings with much more uncertainties than that of China. Second, the mathematical model is a deterministic model which cannot capture stochastic effects during the spread of COVID-19 under the influence of multiple socio-environmental factors. This may bias the number of incident cases of a modelled scenario. Third, we considered municipal cities as the spatial components, however, these publically available data were sparse at the city level for modelling a city-specific reproduction number, and therefore we assumed it the same across all cities. Although that the reproduction number for the entire country we calculated in this study is not substantially different from the others using a general SEIR model, it is possible that the transmissibility of COVID-19 might change from place to place. The basic reproduction number in the current setting could be underestimated, especially when the restricted travels of all SEIR individuals would underestimate the number of active cases in each region [24]. Considering that fewer people travel making each region behaving as epidemiologically isolated region in the presence of lockdown, future studies would focus on the identification of hot-spot cities at risks of becoming endemic allowing for geographical variation in reproduction number estimation. Fourth, the three simulated scenarios were based on different restrictive measures of population mobility, lacking control of other confounding factors, and therefore results should be interpreted with caution.

## Conclusion

Seventeen years after the Severe Acute Respiratory Syndrome (SARS) epidemic, the current COVID-19 pandemic serves as a reminder of how rapidly novel pathogens could appear and spread across the nation and in the world with devastating consequences. Our results strongly supported the travel restriction as one of the emergency responses to this global population health threat, provided evidence that *chunyun* was a stimulus of the geographic spread of COVID-19 in China, and highlighted the importance of developing area-specific countermeasures. The simulations suggested continuing restriction on population mobility where appropriate help in prevention and control of the COVID-19 pandemic. This was in particular relevance to any receiving cities in China and many other countries around the world that faced massive inter-city travel demands.

## Data Availability

Provincial Health Commissions in mainland of China have reported municipal-level incident numbers of COVID-19 suspected, confirmedly infected, recovered, and deceased individuals, respectively on a daily basis since January 2020 (National Health Commission of China. Daily updates on the pneumonia epidemic situation. http://www.nhc.gov.cn/xcs/yqtb/list_gzbd.shtml). Public access to this daily data release and update is open as at April 2, 2021. Baidu Migration Index data are publically available and could be obtained from https://qianxi.baidu.com/. Data from this study are available from the authors upon reasonable request.

## Abbreviations

CDC: Center for Disease Control and Prevention
COVID-19: COrona VIrus Disease 2019
SARS: Severe Acute Respiratory Syndrome
